# 35336 Effect of Nuclear Soluble Adenylyl Cyclase (sAC) on Melanoma Treatment Response

**DOI:** 10.1017/cts.2021.754

**Published:** 2021-03-30

**Authors:** Jakyung Bang, Marek M. Drozdz, Lauren Dong, Taha Merghoub, Jonathan H. Zippin

**Affiliations:** 1 Joan and Sanford I. Weill Medical College of Cornell University; 2 Memorial Sloan Kettering Cancer Center

## Abstract

ABSTRACT IMPACT: Our data identify a novel candidate for combination strategy in melanoma treatment, and can inform clinicians in their decision-making process regarding therapeutic intervention for melanoma patients. OBJECTIVES/GOALS: Soluble adenylyl cyclase (sAC) is a novel source of cyclic AMP (cAMP). In melanoma, nuclear sAC localization has an established diagnostic utility and we newly found that nuclear sAC functions as a tumor suppressor by inhibiting Hippo pathway, which affects treatment response. Here, we examine the effect of nuclear sAC on melanoma treatment response. METHODS/STUDY POPULATION: We developed a doxycycline inducible system for increasing sAC activity only in the nucleus. We assessed whether nuclear sAC activity affects treatment response, using BRAFV600 human melanoma cell lines. Using a clonogenic assay, we examined how nuclear sAC activity affects growth inhibition in the presence of a BRAF inhibitor, vemurafenib. Our findings will be confirmed in vivo using tumor xenografts. After tumor formation in NSG mice, mice will be randomized to be fed normal or doxycycline chow for nuclear sAC induction, then subdivided to receive vehicle or vemurafenib to examine the effect of nuclear sAC activity on treatment response in vivo. We will also compare melanoma biopsies collected before and after treatment with BRAF inhibitors to assess how nuclear sAC staining affects tumor morphology in vivo. RESULTS/ANTICIPATED RESULTS: So far, nuclear sAC activity has rendered SkMel178 and M263 cell lines more susceptible to vemurafenib. Cell viability was inversely correlated both with vemurafenib and with doxycycline concentration. Cell viability after vemurafenib treatment was dramatically reduced when nuclear sAC was activated. It appears that nuclear sAC enhances the sensitivity of BRAF mutant melanomas to vemurafenib in vitro. We anticipate that xenografts of these cells in mice will be more susceptible to vemurafenib when nuclear sAC is activated. We also anticipate that positive nuclear sAC staining will correlate with a favorable response to therapy. DISCUSSION/SIGNIFICANCE OF FINDINGS: Targeted therapy with BRAF inhibitors is used in late-stage melanomas, but its use is limited as patients invariably acquire resistance. Here, we identified nuclear sAC activation as a novel candidate for combination strategy. Our data will also inform clinicians how best to integrate this biomarker into their decision-making regarding therapy.

